# A selective histone deacetylase-6 inhibitor improves BDNF trafficking in hippocampal neurons from *Mecp2* knockout mice: implications for Rett syndrome

**DOI:** 10.3389/fncel.2014.00068

**Published:** 2014-03-07

**Authors:** Xin Xu, Alan P. Kozikowski, Lucas Pozzo-Miller

**Affiliations:** ^1^Department of Neurobiology, Civitan International Research Center, The University of Alabama at BirminghamBirmingham, AL, USA; ^2^Drug Discovery Program, Department of Medicinal Chemistry and Pharmacognosy, University of Illinois at ChicagoChicago, IL, USA

**Keywords:** Rett syndrome, dense core vesicle, activity-dependent BDNF release, Tubastatin-A, tubulin acetylation

## Abstract

Rett syndrome (RTT) is a neurodevelopmental disorder caused by loss-of-function mutations in the transcriptional modulator methyl-CpG-binding protein 2 (*MECP2*). One of the most prominent gene targets of MeCP2 is brain-derived neurotrophic factor (*Bdnf*), a potent modulator of activity-dependent synaptic development, function and plasticity. Dysfunctional BDNF signaling has been demonstrated in several pathophysiological mechanisms of RTT disease progression. To evaluate whether the dynamics of BDNF trafficking is affected by *Mecp2* deletion, we analyzed movements of BDNF tagged with yellow fluorescent protein (YFP) in cultured hippocampal neurons by time-lapse fluorescence imaging. We found that both anterograde and retrograde vesicular trafficking of BDNF-YFP are significantly impaired in *Mecp2* knockout hippocampal neurons. Selective inhibitors of histone deacetylase 6 (HDAC6) show neuroprotective effects in neurodegenerative diseases and stimulate microtubule-dependent vesicular trafficking of BDNF-containing dense core vesicles. Here, we show that the selective HDAC6 inhibitor Tubastatin-A increased the velocity of BDNF-YFP vesicles in *Mecp2* knockout neurons in both directions by increasing α–tubulin acetylation. Tubastatin-A also restored activity-dependent BDNF release from *Mecp2* knockout neurons to levels comparable to those shown by wildtype neurons. These findings demonstrate that a selective HDAC6 inhibitor is a potential pharmacological strategy to reverse cellular and synaptic impairments in RTT resulting from impaired BDNF signaling.

## Introduction

Rett syndrome (RTT), an X-linked postnatal neurodevelopmental disorder associated with intellectual disabilities, is primarily caused by mutations in methyl-CpG-binding protein 2 (*MECP2*), the gene encoding MeCP2, a transcriptional modulator that binds to methylated CpG sites in promoter regions of DNA (Nan et al., 1997; Amir et al., 1999; Percy and Lane, 2005). A number of genes including brain-derived neurotrophic factor (*Bdnf*) were identified to be regulated by MeCP2 and relevant to the pathogenesis of RTT (Bievenu and Chelly, 2006; Chahrour and Zoghbi, 2007). MeCP2 binds to the *Bdnf* promoter and directly modulates *Bdnf* expression in an activity-dependent manner (Chen et al., 2003; Martinowich et al., 2003; Zhou et al., 2006). Several studies have reported lower BDNF mRNA and protein levels in various brain regions of *Mecp2* deficient mice and RTT individuals (Chang et al., 2006; Wang et al., 2006; Ogier et al., 2007; Li et al., 2012). Reduced overall neuronal activity caused by MeCP2 deficiency is thought to contribute to BDNF downregulation. Conditional *Bdnf* mutant mice showed similar RTT phenotypes as *Mecp2* knockout mice, while *Bdnf* overexpression rescued some of the functional deficits observed in *Mecp2* mutants and extended their lifespan (Chang et al., 2006; Chahrour and Zoghbi, 2007). These findings strongly indicate BDNF plays a critical role in neurological dysfunctions in RTT.

Prior to RTT, BDNF had been implicated in other neurological disorders due to its widespread function in neuronal development, plasticity, differentiation, and survival (Poo, 2001; Fahnestock et al., 2002; Gines et al., 2010; Hartmann et al., 2012). Common among these BDNF-related disorders, such as Alzheimer's disease (AD), Huntington disease (HD), is the irregular trafficking of dense-core vesicles containing BDNF, as well as activity-dependent BDNF release from those vesicles (Gauthier et al., 2004; Chapleau et al., 2009; Poon et al., 2011). Intriguingly, the single nucleotide polymorphism Val66Met observed in the human *BDNF* gene resulted in more severe RTT symptoms and an increased risk of seizure onset, suggesting that in addition to BDNF expression levels, BDNF trafficking and release are altered in RTT (Zeev et al., 2009; Hartmann et al., 2012). Live BDNF-YFP imaging in cultured neurons offers the ability to investigate dynamic trafficking of BDNF, which was reported to be identical to that of endogenous BDNF in terms of its cellular localization, processing and secretion (Haubensak et al., 1998; Kohara et al., 2001; Lessmann and Brigadski, 2009; Hartmann et al., 2012). Here, we report that vesicular trafficking of BDNF, as well as its activity-dependent release are significantly impaired in hippocampal neurons of *Mecp2* knockout mice, providing further support for the role of BDNF signaling in RTT pathophysiology.

Histone deacetylase-6 (HDAC6), a member of the class II histone deacetylases, is a unique cytosolic enzyme that regulates cell motility (Hubbert et al., 2002; Matsuyama et al., 2002; Zhang et al., 2003; Tran et al., 2007), endocytosis (Gao et al., 2007), vesicle transport (Dompierre et al., 2007), cell migration and degradation of misfolded proteins (Iwata et al., 2005; Valenzuela-Fernandez et al., 2008) and other cellular process by deacetylating α-tubulin, Hsp90 and cortactin (Fukada et al., 2012). HDAC6 has emerged as an attractive target for pharmacological intervention in several CNS diseases. Selective inhibition of HDAC6 is thought to promote neuronal survival and regrowth after injury, offering a potential therapy for various neurodegenerative diseases (Kazantsev and Thompson, 2008; Rivieccio et al., 2009; Butler et al., 2010). For example, the non-selective HDAC inhibitor trichostatin A (TSA) improves microtubule (MT)-dependent transport of BDNF-GFP in cultured neurons expressing mutant Huntingtin; this effect was ascribed to increased α–tubulin acetylation through the inhibition of cytoplasmic HDAC6 (Dompierre et al., 2007). Indeed, Tubastatin-A (TBA), a more selective HDAC6 inhibitor, showed neuroprotective effects in a model of oxidative stress, and exhibited no toxicity compared to TSA (Butler et al., 2010). Furthermore, TBA rescued the impairment of mitochondrial transport in axons and mitochondrial elongation caused by Aβ exposure (Kim et al., 2012). We report that TBA improves BDNF-YFP trafficking and activity-dependent release in *Mecp2* knockout hippocampal neurons to reach wildtype levels, suggesting that HDAC6 is a potential therapeutic target to restore BDNF-dependent neurological function in the absence of functional MeCP2, which provides a novel approach for therapeutic intervention in RTT.

## Materials and methods

### Animals

Breeding pairs of mice lacking exon 3 of the X chromosome-linked *Mecp2* gene (B6.Cg-*Mecp2*^tm1.1Jae^, “Jaenisch” strain in a pure C57BL/6 background) (Chen et al., 2001) were purchased from the Mutant Mouse Regional Resource Center at the University of California, Davis. A colony was established at The University of Alabama at Birmingham (UAB) by mating wildtype males with heterozygous *Mecp2*^tm1.1Jae^ mutant females, as recommended by the supplier. Genotyping was performed by PCR of DNA sample from tail clips. Hemizygous *Mecp2*^tm1.1Jae^ mutant males are healthy until 5–6 weeks of age, when they exhibit RTT-like motor symptoms, such as hypoactivity, hind limb clasping, and reflex impairments (Chen et al., 2001). Animals were handled and housed according to the Committee on Laboratory Animal Resources of the National Institutes of Health. All experimental protocols were annually reviewed and approved by the Institutional Animals Care and Use Committee of UAB.

### Primary cultures of hippocampal neurons and transfection

Both hippocampi were dissected from anesthetized postnatal day 0 or 1 (P0-1) male *Mecp2* knockout mice and wildtype littermates, and dissociated in papain (20 U/ml) plus DNAse I (Worthington, Lakewood, NJ) for 20-30 min at 37°C, as described (Amaral and Pozzo-Miller, 2007). The tissue was then triturated to obtain a single-cell suspension, and the cells were plated at a density of 50,000 cells/cm^2^ on 12 mm poly-L-lysine/laminin coated glass coverslips, and immersed in Neurobasal medium supplemented with 2% B27 and 0.5 mM glutamine (Life Technologies, Carlsbad, CA). Neurons were grown in 37°C, 5% CO_2_, 90% relative humidity incubators (Thermo-Forma), with half of the fresh medium changed every 3-4 days. After 11 days *in vitro* (DIV), neurons were transfected with cDNA encoding BDNF-YFP (a gift from M. Kojima) using Lipofectamine 2000 (Life Technologies) (0.8 μg DNA) according to the manufacturer's protocol.

### Immunocytochemistry

All experiments were performed at 12-14 DIV. For localization of endogenous native BDNF, neurons were fixed with 4% (wt/vol) paraformaldehyde/sucrose in phosphate buffer (PB; 23 mM NaH_2_PO4, 2 mM Na_2_HPO4 pH 7.4) for 10 min, and incubated in 0.25% (vol/vol) Triton X-100 for 10 min, then washed with PB saline (PBS). After blocking with 10% (vol/vol) goat serum in PBS, cells were incubated with anti-BDNF antibody (2μ g/ml, Santa Cruz Biotechnology #SC-546) overnight at 4°C, rinsed in PBS, and incubated with Alexa Fluor-488 secondary antibody (Life Technologies) for 1 h; coverslips were then mounted with Vectashield (Vector Laboratories). Images were acquired in a laser-scanning confocal microscope using a solid-state 488 nm laser for excitation, and a 60 X 1.4 NA oil immersion lens, and standard FITC dichroic and emission filters (FluoView-300, Olympus; Center Valley, PA). For dual immunocytochemistry, BDNF-YFP-expressing neurons were fixed with 3% formaldehyde in PB at 0°C for 20 min, permeabilized for 10 min at room temperature with 3% formaldehyde containing 0.25% Triton X-100, and blocked with 10% bovine serum albumin (BSA) for 1 h at 37°C. Primary goat anti-chromogranin B (1:100; Santa Cruz) and rabbit anti-BDNF (1:100, Santa Cruz) antibodies, as well as fluorescently-conjugated anti-goat and anti-rabbit secondary antibodies (Molecular Probes) were diluted in 1X PBS and 3% horse serum. Coverslips were mounted with Vectashield (Vector Laboratories), and fluorescence images were acquired with a cooled CCD camera (CoolSnap HQ2, Photometics) on a wide-field fluorescence microscope (Eclipse TE2000-U, Nikon Instruments); BDNF-YFP was imaged with a standard FITC filter, and fluorescently-conjugated secondary antibodies with standard FITC and TRITC filters. Images were deconvolved using Metamorph (Molecular Devices).

### Time-lapse fluorescence imaging

Time-lapse imaging was performed 24–48 h after BDNF-YFP transfection. For mitochondria trafficking, neurons were incubated for 15 min with MitoTracker Red (200 nM; Life Technologies) prior to live imaging. Individual coverslips with cultured neurons were transferred to a recording chamber mounted on a fixed-stage upright microscope (Leica DM-LFS with either a 63 × 0.9 NA or a Zeiss 63 × 1.0 NA water-immersion objective), and continuously perfused (1 ml/min) with HEPES buffered artificial CSF (aCSF) at 32–34°C, containing (mM): 119 NaCl, 5 KCl, 2 CaCl_2_, 1.3 MgCl_2_, 10 glucose, 10 HEPES (pH 7.4). YFP was excited with 490 ± 12 nm light using a galvanometric monochromator (Polychrome-II, TILL Photonics; Germany), and its emission (>510 nm, FITC-LP cube) was filtered and detected with an electron-multiplying cooled CCD camera operating in frame-transfer mode (QuantEM: 512SC, Photometrics, Tucson AZ). MitoTracker Red was excited with 560 ± 12 nm and imaged through a standard TRITC cube. Digital images were acquired every 5 s (50–100 ms exposures for ~100 × 200 pixel sub-arrays, 1 × 1 binning) for a total time of 10 min. The position of fluorescent puncta was tracked as a function of time using the Particle Tracking module of Imaris (Bitplane).

### Drug treatments and cell viability assays

BDNF-YFP-transfected neurons were treated with the HDAC6 inhibitor Tubastatin A (1 μM, prepared in 0.01% DMSO vehicle) for 48 h; 0.01% DMSO was used as control. Cell viability was assessed by trypan blue exclusion. Cells were rinsed with HBSS, and incubated with 0.4% trypan blue solution (Gibco) for 2 min at room temperature. After washing with HBSS, dead and live cells were counted in ten random fields per coverslip.

### Surface staining of BDNF-YFP immunofluorescence

The procedure for BDNF-YFP immunostaining on the surface of cultured neurons was as described (Sadakata et al., 2012). Twenty-four hours after BDNF-YFP transfection, cultured neurons were stimulated with 50 mM KCl for 10 min in the absence or presence of the voltage-gated Ca^2+^ channel blocker nifedipine (50 μ M), the ionotropic glutamate receptor antagonists CNQX (10 μ M) and D-APV (50 μ M), the GABA_A_R antagonist bicuculline (10 μ M), or the voltage-gated Na^+^ channel blocker TTX (0.5 μ M), fixed with 4% (wt/vol) paraformaldehyde/sucrose for 5 min, and then washed with PBS. After blocking with 10% (vol/vol) goat serum in PBS, cells were incubated with anti-GFP antibody (which also recognized YFP; Abcam) overnight at 4°C, rinsed in PBS, and incubated with anti-rabbit secondary antibody conjugated to Cy3 (Millipore) for 1 h; coverslips were then mounted with Vectashield medium (Vector Laboratories). Images were acquired in a laser-scanning confocal microscope using a solid-state 488 nm laser for YFP excitation, a 543 nm HeNe green laser for Cy3 excitation, a 60 × 1.4 NA oil immersion lens, and standard FITC and TRITC dichroic and emission filters (FluoView-300, Olympus). Quantification of co-localized pixels was performed using Colocalization module in Imaris. The ratio of surface-bound BDNF-YFP to total BDNF-YFP was estimated as volumetric percentage of co-localized signals over the threshold.

### Western immunoblotting

For biochemical measurements of the levels of acetylated and total α-tubulin, total cell lysates were obtained by rinsing 4 × 10^5^ treated hippocampal neurons with cold PBS followed by lysis in NP-40 lysis buffer (20 mM Tris at pH 8.0, 137 mM NaCl, 10% glycerol, 1% Nonidet P-40, 2 mM EDTA) containing protease inhibitor. The cell lysates were maintained with constant agitation for 30 min at 4°C and centrifuged at 12,000 g for 20 min. The supernatants were aspirated and protein concentrations were quantified by the Lowry method. Fifteen micrograms of total lysate were subjected to SDS-PAGE (Bio-Rad) and Western blot analysis with primary antibodies against acetylated α-tubulin (1:1000; Sigma) and total α-tubulin (1:2000; Life Technologies). Immunodetection was performed using Odyssey infrared imaging system (Li-Cor Bioscience).

### Statistical analyses

All the experiments were performed at least 3 different times, from at least 3 different neuronal culture preparations. Data are presented as mean ± standard error of the mean (SEM), and were compared using unpaired Student's *t*-test for two groups or One Way ANOVA with Tukey post test for more than three groups; percentages were compared using Chi-square test. All analyses were performed using Prism software (GraphPad Software, San Diego, CA). *P* < 0.05 was considered significant.

## Results

### BDNF-YFP trafficking is impaired in *Mecp2* knockout neurons

**Figure 1:**
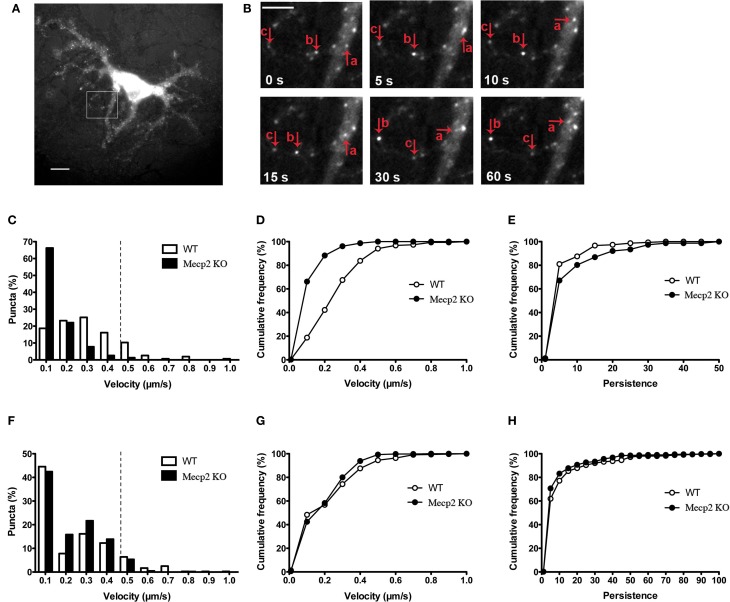
**BDNF-YFP trafficking is impaired in *Mecp2* knockout neurons**. **(A)** Representative example of a cultured pyramidal neuron expressing BDNF-YFP; scale bar = 10 μm. The white square is enlarged in **(B)**. **(B)** Movement of BDNF-YFP punta (a, b, and c); scale bar = 5 μm. Each image was taken at the time point indicated at bottom left. **(C)** Distributions of velocities of BDNF-YFP puncta in wildtype and *Mecp2* knockout neurons. **(D)** Cumulative frequency of velocities of BDNF-YFP puncta from the histograms shown in **(C)**. **(E)** Cumulative frequency of persistence of BDNF-YFP puncta. **(F)** Distributions of velocities of MitoTracker-Red puncta in wildtype and *Mecp2* knockout neurons. **(G)** Cumulative frequency of velocities of MitoTracker-Red puncta from the histograms shown in **(F)**. **(H)** Cumulative frequency of persistence of MitoTracker-Red puncta.

The cellular localization, processing and secretion of exogenously expressed BDNF-xFP have been reported to be identical to those of endogenous native BDNF, including its co-localization to secretory granule cargoes like chromogranin-B and SGA2 (Haubensak et al., 1998; Kohara et al., 2001; Lessmann and Brigadski, 2009; Hartmann et al., 2012) (Supplemental Figure 1). To evaluate whether the dynamics of dendritic BDNF trafficking is altered by *Mecp2* deletion, time-lapse fluorescence imaging of BDNF-YFP was performed in primary cultures of hippocampal neurons (12–14 days *in vitro*, DIV) from male *Mecp2* knockouts and wildtype littermates. BDNF-YFP puncta are widely distributed and move bi-directionally in live neurons (Figures 1A,B), as previously described (Park et al., 2008; Matsuda et al., 2009). The average velocity of BDNF-YFP puncta was significantly slower in *Mecp2* knockout neurons (0.10 ± 0.01 μm/s, *n* = 261 puncta from 8 cells) compared to wildtype cells (WT 0.25 ± 0.01 μm/s, *n* = 225 puncta from 8 cells; *p* < 0.001). Analyses of the distributions of the velocity of BDNF-YFP puncta revealed that the number of fast moving puncta (velocity >0.4 μm/s) is significantly smaller in *Mecp2* knockout neurons (*Mecp2* 1.30 % vs. WT 16.77 %; *p* < 0.001; Figures 1C,D). To quantify BDNF transport efficiency, we compared the persistence of each BDNF-YFP puncta, estimated as the ratio of total distance traveled during an image sequence over the minimum displacement between image frames (Gauthier et al., 2004). Consistent with impaired and slower BDNF trafficking, the persistence of BDNF-YFP puncta was higher in *Mecp2* knockout neurons (*Mecp2* 7.14 ± 0.98 vs. WT 3.91 ± 0.43; *p* < 0.05; Figure 1E). Then, we tested whether the transport of other organelles such as mitochondria is also affected by *Mecp2* deletion. Intriguingly, time-lapse imaging of MitoTracker-Red puncta revealed a lower percentage of fast moving mitochondria in *Mecp2* knockout neurons (*Mecp2* 6.08 % vs. WT 12.39 %; *p* < 0.01; Figure 1F), but no significant differences in their average velocity or persistence between *Mecp2* knockout (*n* = 410 puncta from 13 cells) and wildtype neurons (*n* = 359 puncta from 14 cells; *p* > 0.05; Figures 1G,H).

### The impairment of BDNF-YFP trafficking in *Mecp2* knockout neurons is bi-directional

**Figure 2:**
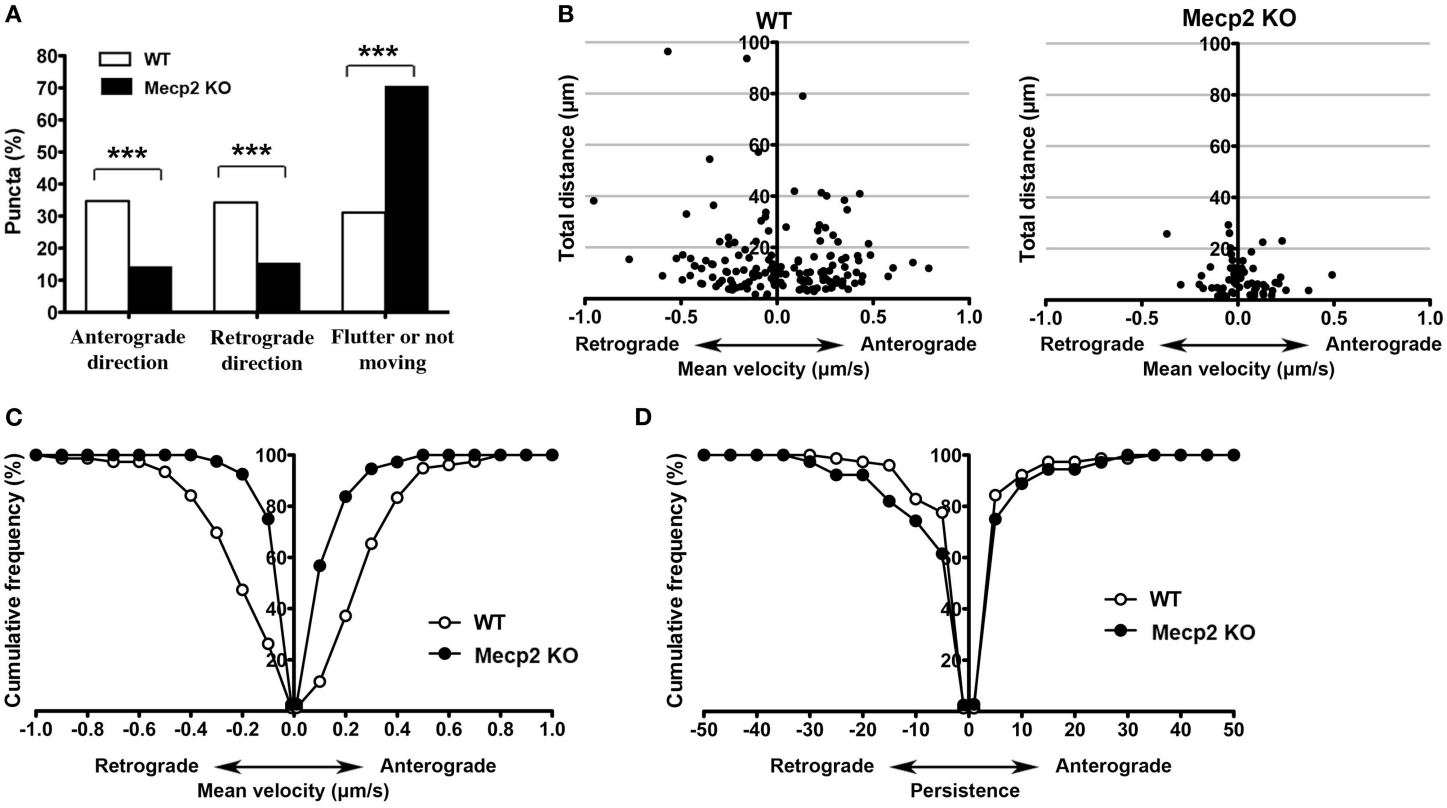
**Bi-directional BDNF-YFP trafficking is impaired in *Mecp2* knockout neurons. (A)** Proportion of BDNF-YFP puncta that moves in the anterograde or retrograde directions, flutter within a small distance (10 μm) or not moving at all in wildtype and *Mecp2* knockout neurons. **(B)** Scatter plots showing analyses of movement of BDNF-YFP puncta in anterograde and retrograde directions. **(C)** Average velocity of BDNF-YFP puncta was significantly slower in *Mecp2* knockout neurons for both directions. **(D)** Persistence of BDNF-YFP puncta was also higher in *Mecp2* knockout neurons for both directions. ^***^p < 0.001 wildtype vs. *Mecp2* knockout.

Anterograde BDNF transport from somata to dendrites and axon terminals is important for activity-dependent BDNF release to participate in synaptic plasticity, while retrograde BDNF transport back to the soma is critical for neurotrophin recycling and nuclear signaling (Egan et al., 2003; Park et al., 2008). When BDNF-YFP puncta were separated by their trafficking direction, *Mecp2* knockout neurons showed significantly fewer BDNF puncta moving in both anterograde and retrograde directions than wildtype neurons, with a significantly shorter total distance traveled in either direction in *Mecp2* knockout cells (*p* < 0.001; Figures 2A,B). Consistently, the average velocity of BDNF-YFP puncta moving in both anterograde and retrograde directions was significantly slower (*p* < 0.001; Figure 2C), and their persistence higher (*p* < 0.05 in retrograde direction; Figure 2D), in *Mecp2* knockout neurons.

### The HDAC6-selective inhibitor TBA improves bi-directional BDNF-YFP trafficking in *Mecp2* knockout neurons

HDAC6 removes acetyl groups from α-tubulin (Hubbert et al., 2002; Matsuyama et al., 2002; Zhang et al., 2003), which makes microtubules less stable. Since BDNF is transported along microtubules (Gauthier et al., 2004), inhibiting HDAC6 is expected to improve BDNF trafficking by stabilizing microtubules (Dompierre et al., 2007). We tested whether treatment with the selective HDAC6 inhibitor enhances BDNF-YFP trafficking in cultured hippocampal neurons. Treatment with Tubastatin A for 48 h (TBA, 1 μM, prepared in 0.01% DMSO) was not neurotoxic, as determined by trypan blue exclusion (*p* > 0.05; Supplemental Figure 2A). Then, we confirmed that TBA affected the acetylation state of α-tubulin under our experimental conditions. Western immunoblots of cultured hippocampal neurons (DIV12) treated with TBA showed that the levels of acetylated tubulin were significantly increased in wildtype neurons (*p* < 0.01; Supplemental Figure 2B), and elevated by two times in *Mecp2* knockout neurons (*p* < 0.001; Supplemental Figure 2B).

**Figure 3:**
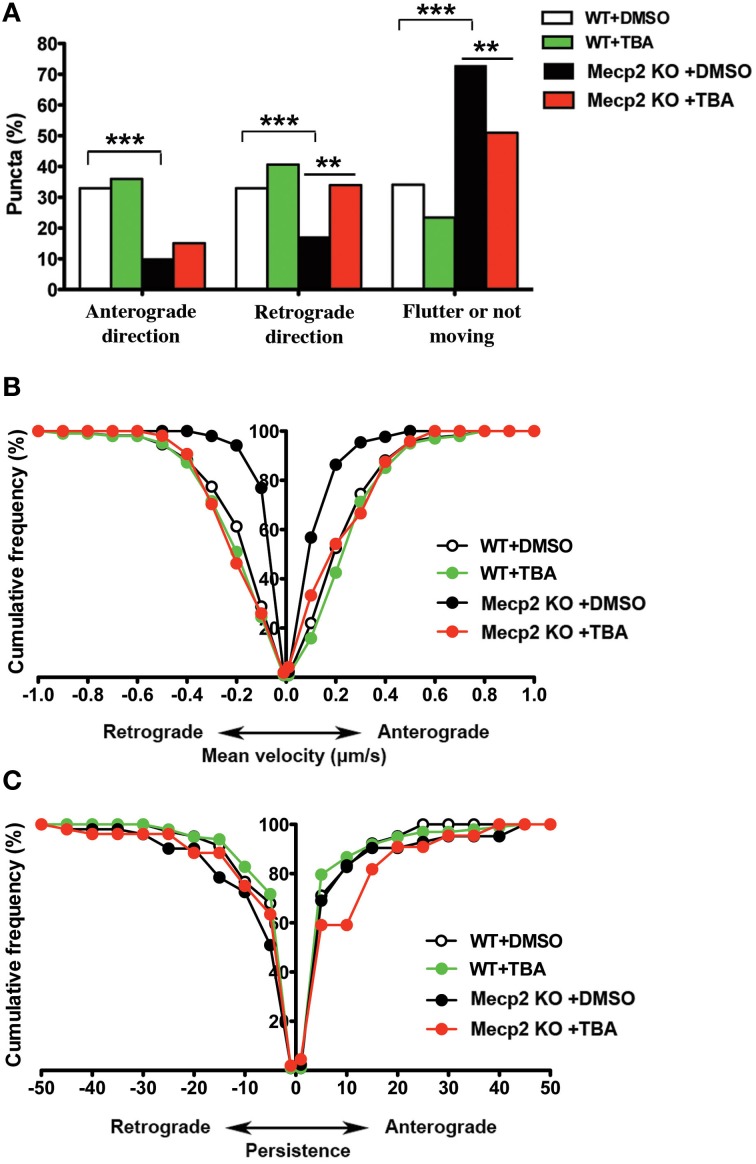
**TBA improves BDNF-YFP trafficking in *Mecp2* knockout neurons. (A)** Proportion of BDNF-YFP puncta that moves in anterograde or retrograde directions, flutter or not move in wildtype and *Mecp2* knockout neurons, with or without TBA. **(B)** TBA increased the average velocity of both anterograde and retrograde BDNF-YFP puncta only in *Mecp2* knockout neurons. **(C)** TBA did not affect BDNF puncta persistence in neither wildtype nor *Mecp2* knockout neurons. ^**^p < 0.01; ^***^p < 0.001 wildtype vs. *Mecp2* knockout.

Next, we tested the effect of HDAC6 inhibition on BDNF-YFP trafficking. Compared to vehicle-treated neurons (162 puncta from 7 cells), TBA increased the proportion of retrogradely moving BDNF puncta (159 puncta from 7 cells; *p* < 0.01; Figure 3A), as well as the average velocity of puncta moving anterogradely and retrogradely in *Mecp2* knockout neurons (*p* < 0.001; Figure 3B). However, TBA did not affect the persistence of BDNF puncta in *Mecp2* knockout neurons (*p* > 0.05; Figure 3C). On the other hand, TBA had no effect in any of these BDNF-YFP trafficking parameters in wildtype neurons (DMSO 106 puncta from 5 cells; TBA 149 puncta from 7 cells; *p* > 0.05; Figures 3A–C).

### Activity-dependent BDNF release was impaired in *Mecp2* knockout neurons, and improved by inhibiting HDAC6

**Figure 4:**
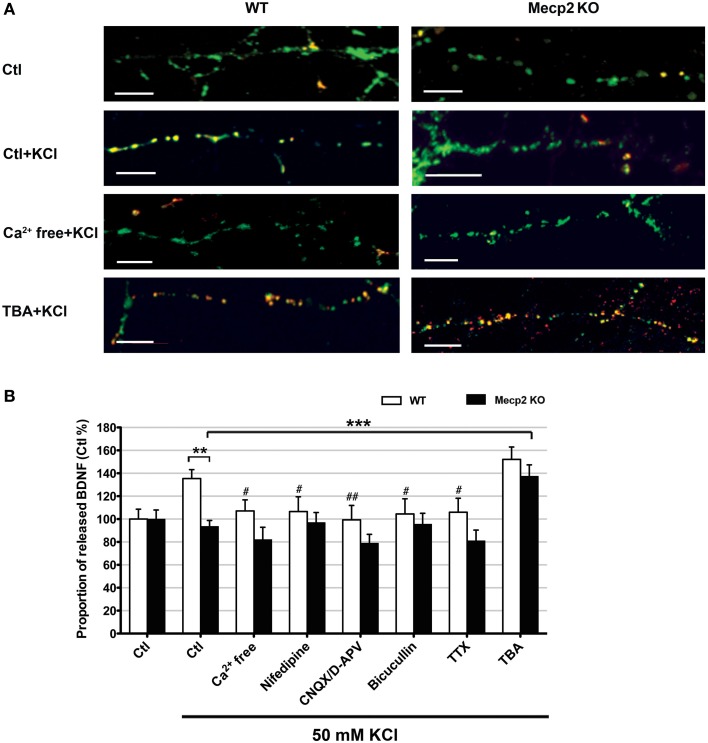
**Activity-dependent release of BDNF-YFP is impaired in *Mecp2* knockout neurons**. **(A)** Representative examples of BDNF release in wildtype and *Mecp2* knockout neurons: no treatment, stimulated with KCl, stimulated with KCl in the absence of extracellular Ca^2+^ and stimulated with KCl upon TBA treatment; scale bar = 10 μm. BDNF-YFP is shown in green, while YFP immunoreactivity is shown in red. Yellow pixels represent colocalization of green and red pixels, i.e., secreted BDNF. **(B)** Proportion of released BDNF per total BDNF upon KCl stimulation in wildtype neurons and *Mecp2* knockout neurons in the absence or presence of extracellular Ca^2+^, nifedipine (50 μ M), CNQX (10μ M) /D-APV (50 μ M), Bicuculline (10 μ M), TTX (0.5 μ M) or TBA (1 μ M). The proportion of released BDNF per total BDNF was normalized to that in non-stimulated neurons (Ctl), and expressed as % of Ctl. ^**^p < 0.01; wildtype vs. *Mecp2* knockout; ^***^p < 0.001 compared to *Mecp2* knockout Ctl group stimulated with KCl; ^#^*p* < 0.05; ^##^*p* < 0.01 compared to wildtype Ctl group stimulated with KCl.

**Figure 5:**
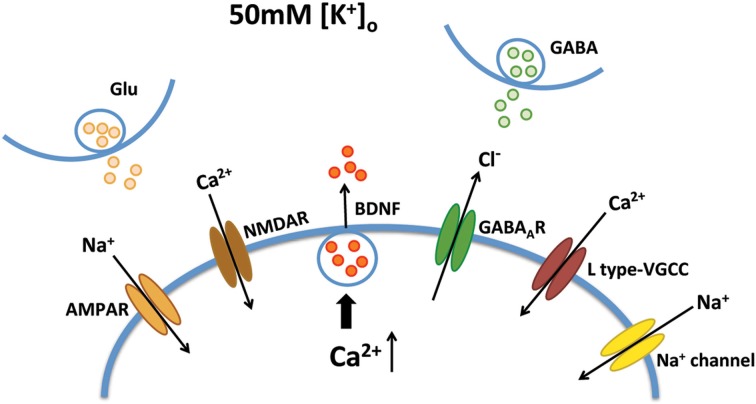
**Mechanisms of Ca^2+^-dependent BDNF release during KCl-induced depolarization**. KCl-induced BDNF secretion depends on Ca^2+^ influx, which can be mediated by L type-VGCC, AMPA receptors, NMDA receptors, GABA_A_ receptors and voltage-sensitive Na^+^ channels.

Improving BDNF transport to release sites could in principle also enhance its activity-dependent release. To estimate activity-dependent BDNF secretion, BDNF-YFP-expressing neurons were depolarized with 50 mM KCl, followed by immunostaining with anti-YFP antibody before cell permeabilization. The degree of co-colocalization between immunopositive puncta (labeled with red-conjugated anti-YFP secondary antibody), and native YFP fluorescent puncta after fixation is directly proportional to BDNF-YFP secreted during neuronal depolarization (Figure 4A), as described (Sadakata et al., 2012). KCl-induced postsynaptic secretion of BDNF depends on Ca^2+^ influx (Hartmann et al., 2001; Kolarow et al., 2007), mainly through L-type voltage-gated Ca^2+^ channels (Wang et al., 2002). Here, we confirmed that elevated KCl-induced BDNF secretion was absent in a Ca^2+^ free solution (*n* = 18 cells), or in the presence of the L-type Ca^2+^ channel blocker nifedipine (50 μM; *n* = 16; *p* < 0.05; Figure 4B). Ca^2+^ channels can also be activated by depolarization from GABA_A_ receptor activity in immature neurons with high intracellular Cl^−^ concentration (Fiorentino et al., 2009; Porcher et al., 2011). Indeed, the GABA_A_ receptor antagonist bicuculline also prevented KCl-induced BDNF secretion (10 μM; *n* = 15; *p* < 0.05; Figure 4B). In addition, KCl-induced BDNF secretion required functional ionotropic glutamate receptor activity (10 μM CNQX, 50 μM D-APV; *n* = 17; *p* < 0.01), and voltage-sensitive Na^+^ channels (0.5 μM TTX; *n* = 19; *p* < 0.05; Figure 4B), as previously described (Lessmann et al., 2003). Figure 5 illustrates the different mechanisms leading to Ca^2+^-dependent BDNF release during KCl-induced depolarization.

Consistent with reports of impaired activity-dependent BDNF release using different assays (Li et al., 2012), the proportion of BDNF secreted during KCl-induced depolarization was significantly smaller in *Mecp2* knockout neurons (*n* = 21) than in wildtype neurons (*n* = 25; *p* < 0.01; Figure 4B). In addition, there was no evidence of BDNF secretion in *Mecp2* knockout neurons under any of the conditions of activity blockade described above (Ca^2+^ free *n* = 15; nifedipine *n* = 13; CNQX/D-APV *n* = 16; bicuculline *n* = 15; TTX *n* = 13; *p* > 0.05; Figure 4B). Intriguingly, TBA significantly improved the proportion of BDNF secreted during KCl-induced depolarization in *Mecp2* knockout neurons (*n* = 14; *p* < 0.001; Figure 4B), but not in wildtype neurons (*n* = 22; *p* > 0.05; Figure 4B). These results suggest that improved microtubule-dependent trafficking allowed a larger pool of BDNF to be transported to release sites and be available for activity-dependent release, underscoring the potential therapeutic benefit of this approach to restore BDNF signaling in RTT.

## Discussion

Dysfunctional BDNF signaling likely contributes to several pathophysiological mechanisms of RTT. Previous studies have reported lower BDNF mRNA and protein levels in *Mecp2*-deficient mice and RTT individuals (Chang et al., 2006; Wang et al., 2006; Abuhatzira et al., 2007). Also, axonal transport of BDNF is altered when *Mecp2* levels are modified in cultured cortical neurons (Roux et al., 2012). Here, we demonstrate impaired bi-directional trafficking of BDNF in hippocampal neurons from *Mecp2* knockout mice. Since anterograde BDNF trafficking likely reflects delivery to release sites, while retrograde BDNF trafficking represents signaling endosomes directed to the cell nucleus (Egan et al., 2003; Park et al., 2008), our results suggest that both activity-dependent BDNF release, as well as neurotrophin recycling and nuclear signaling are affected in MeCP2-deficient neurons. Using a novel assay of BDNF secretion based on surface immunostanining of BDNF-YFP in live neurons after neuronal depolarization (Sadakata et al., 2012), we confirmed that activity-dependent BDNF release is impaired in hippocampal neurons from *Mecp2* knockout mice (Li et al., 2012). Since microtubules containing acetylated α-tubulin are more stable, and BDNF vesicles are transported along microtubules (Gauthier et al., 2004), we confirmed that selective inhibition of cytoplasmic HDAC6—which increases acetylated α-tubulin levels—improves not only BDNF trafficking, but also its activity-dependent secretion. Taken together, our findings suggest that, in addition to BDNF mRNA and protein levels, dysfunctional BDNF trafficking and release contribute to RTT neuropathology. Thus, targeting the molecular machinery responsible for BDNF trafficking and release represents a novel strategy to reverse BDNF-dependent neurological deficits in RTT.

Is this impairment in trafficking specific for BDNF-containing vesicles, or does it affect other cellular elements transported along microtubules? *Mecp2* deficiency impairs axonal transport of amyloid precursor protein (App) without affecting its mRNA and protein levels (Roux et al., 2012). Also, we showed here that *Mecp2* knockout neurons have fewer fast-moving mitochondria than wildtype neurons, suggesting that MeCP2 deficiency affects microtubule-dependent transport in general. Whether and how the molecular motors responsible for anterograde (kinesin-1), and retrograde transport (dynein/dynactin) are affected in RTT needs to be further explored.

We showed that the selective HDAC6 inhibitor TBA improves BDNF trafficking in *Mecp2* knockout neurons by increasing the proportion of moving BDNF puncta and their average velocity in both anterograde and retrograde directions. TBA also restored activity-dependent BDNF release in *Mecp2* knockout neurons to levels comparable to those of wildtype neurons. How did HDAC6 inhibition produce these effects? It is known that acetylation of α-tubulin at Lys-40 promotes axonal transport of cargo proteins by increasing microtubule stability (Reed et al., 2006; Bulinski, 2007), and that inhibition of HDAC6 is responsible for such increased α-tubulin acetylation that leads to enhanced axonal transport of lysosomes and other secretory vesicles (Dompierre et al., 2007). Consistently, *in vivo* treatment with TBA improves axonal trafficking in a mouse model of Charcot-MarieTooth disease by increasing α-tubulin acetylation and enhancing microtubule rigidity (d'Ydewalle et al., 2011). Therefore, HDAC6 inhibitors represent a potential therapeutic strategy for neurodegenerative disorders in which microtubule-dependent intracellular transport is impaired (Dompierre et al., 2007; Kim et al., 2012). Our results also showed that TBA increased α-tubulin acetylation in wildtype neurons, but not as much as in *Mecp2* knockout neurons. However, we didn't find any effect of TBA in wildtype neurons. This might be because BDNF trafficking is in the optimal state in wildtype neurons. TBA restored impaired BDNF trafficking in *Mecp2* knockout neurons to wildtype levels.

In the present study with BDNF-YFP transfected in hippocampal neurons, we cannot exclude an artifact of BDNF overexpression to endogenous levels. However, the intracellular localization of BDNF-YFP is similar to that of endogenous BDNF, including its co-localization to secretory granule cargoes like chromogranin-B and SGA2 (Haubensak et al., 1998; Kohara et al., 2001; Lessmann and Brigadski, 2009; Hartmann et al., 2012) (Supplemental Figure 1), suggesting that the trafficking properties of BDNF-YFP can be comparable to those of endogenous BDNF. Even though BDNF expression is higher, we cannot ignore the differences of BDNF trafficking and release between wildtype and *Mecp2* knockout neurons.

In conclusion, our findings revealed that bi-directional trafficking of BDNF and its activity-dependent release are significantly impaired in hippocampal neurons of *Mecp2* knockout mice, and that this deficit can be improved by enhancing tubulin acetylation with a selective HDAC6 inhibitor, which should improve microtubule-based transport. Targeting molecular components responsible for microtubule-based trafficking of BDNF-containing dense core vesicles is a potential strategy to reverse cellular and synaptic impairments in RTT.

### Conflict of interest statement

The authors declare that the research was conducted in the absence of any commercial or financial relationships that could be construed as a potential conflict of interest.

## Supplementary material

Supplemental Figure 1 **Distribution of exogenously expressed BDNF-YFP is comparable to that of endogenous native BDNF. (A)** Representative example of a cultured hippocampal pyramidal neuron after dual immunostaining for endogenously expressed native BDNF (red) with chromogranin-B (green), a marker of secretory granules. The inset shows colocalization in a dendritic segment. **(B)** Immunostaining for chromogranin-B (red) on a BDNF-YFP-expressing neuron (green) reveals essentially the same patter of colocalization. **(C,D)** The same pattern of co-localization between exogenous BDNF-YFP and endogenous BDNF is observed in cultured hippocampal pyramidal neurons from either WT **(C)** mice or *Mecp2* knockout mice **(D)**. Scale bar = 10 μm.

Supplemental Figure 2 **TBA increases tubulin acetylation. (A)** Cell viability assessed by trypan blue exclusion. **(B)** Extracts from DIV12 wildtype cells and *Mecp2* knockout neurons treated with or without TBA (1 μ M) for 48 h were processed by Western blot and analyzed for acetylated tubulin and total tubulin. GAPDH was performed as loading control (top). Quantification of protein levels for acetylated tubulin in each group (*n* = 4) was normalized to that in wildtype neurons (WT) and expressed as % of WT. ^*^*p* < 0.05; ^**^*p* < 0.01 compared to WT group; ^***^*p* < 0.001 compared to *Mecp2* knockout group (bottom).

## References

[B1] Abuhatzira L. Makedonski K. Kaufman Y. Razin A. Shemer R. (2007). MeCP2 deficiency in the brain decreases BDNF levels by REST/CoREST-mediated repression and increases TRKB production. Epigenetics 2, 214–222 10.4161/epi.2.4.5212 18075316

[B2] Amaral M. D. Pozzo-Miller L. (2007). TRPC3 channels are necessary for brain-derived neurotrophic factor to activate a non-selective cationic current and to induce dendritic spine formation. J. Neurosci. 27, 5179–5189 10.1523/JNEUROSCI.5499-06.2007 17494704 PMC2806846

[B3] Amir R. E. Van den Veyver I. B. Wan M. Tran C. Q. Francke U. Zoghbi H. Y. (1999). Rett syndrome is caused by mutations in X-linked MECP2, encoding methyl-CpG-binding protein 2. Nat. Genet. 23, 185–188 10.1038/13810 10508514

[B4] Bievenu T. Chelly J. (2006). Molecular genetics of Rett syndrome: when DNA methylation goes unrecognized. Nat. Rev. Genet. 7, 415–426 10.1038/nrg1878 16708070

[B5] Bulinski J. C. (2007). Microtubule modification: acetylation speeds anterograde traffic flow. Curr. Biol. 17, R18–R20 10.1016/j.cub.2006.11.036 17208171

[B6] Butler K. V. Kalin J. Brochier C. Vistoli G. Langley B. Kozikowski A. P. (2010). Rational design and simple chemistry yield a superior, neuroprotective HDAC6 inhibitor, tubastatin A. J. Am. Chem. Soc. 132, 10842–10846 10.1021/ja102758v 20614936 PMC2916045

[B7] Chahrour M. Zoghbi H. Y. (2007). The story of Rett syndrome: from clinic to neurobiology. Neuron 56, 422–437 10.1016/j.neuron.2007.10.001 17988628

[B8] Chang Q. Khare G. Dani V. Nelson S. Jaenisch R. (2006). The disease progression of Mecp2 mutant mice is affected by the level of BDNF expression. Neuron 49, 341–348 10.1016/j.neuron.2005.12.027 16446138

[B9] Chapleau C. A. Larimore J. L. Theibert A. Pozzo-Miller L. (2009). Modulation of dendritic spine development and plasticity by BDNF and vesicular trafficking: fundamental roles in neurodevelopmental disorders associated with mental retardation and autism. J. Neurodev. Disord. 1, 185–196 10.1007/s11689-009-9027-6 19966931 PMC2788955

[B10] Chen R. Z. Akbarian S. Tudor M. Jaenisch R. (2001). Deficiency of methyl-CpG binding protein-2 in CNS neurons results in a Rett-like phenotype in mice. Nat. Genet. 27, 327–331 10.1038/85906 11242118

[B11] Chen W. G. Chang Q. Lin Y. Meissner A. West A. E. Griffith E. C. (2003). Derepression of BDNF transcription involves calcium-dependent phosphorylation of MeCP2. Science 302, 885–889 10.1126/science.1086446 14593183

[B12] Dompierre J. P. Godin J. D. Charrin B. C. Cordelières F. P. King S. J. Humbert S. (2007). Histone deacetylase 6 inhibition compensates for the transport deficit in Huntington's disease by increasing tubulin acetylation. J. Neurosci. 27, 3571–3583 10.1523/JNEUROSCI.0037-07.2007 17392473 PMC6672116

[B13] d'Ydewalle C. Krishnan J. Chiheb D. M. Van Damme P. Irobi J. Kozikowski A. P. (2011). HDAC6 inhibitors reverse axonal loss in a mouse model of mutant HSPB1-induced Charcot-Marie-Tooth disease. Nat. Med. 17, 968–974 10.1038/nm.2396 21785432

[B14] Egan M. F. Kojima M. Callicott J. H. Goldberg T. E. Kolachana B. S. Bertolino A. (2003). The BDNF val66met polymorphism affects activity-dependent secretion of BDNF and human memory and hippocampal function. Cell 112, 257–269 10.1016/S0092-8674(03)00035-7 12553913

[B15] Fahnestock M. Garzon D. Holsinger R. M. Michalski B. (2002). Neurotrophic factors and Alzheimer's disease: are we focusing on the wrong molecule? J. Neural Transm. Suppl. 62, 241–252 10.1007/978-3-7091-6139-5_22 12456067

[B16] Fiorentino H. Kuczewski N. Diabira D. Ferrand N. Pangalos M. N. Porcher C. (2009). GABA(B) receptor activation triggers BDNF release and promotes the maturation of GABAergic synapses. J. Neurosci. 29, 11650–11661 10.1523/JNEUROSCI.3587-09.2009 19759312 PMC6665767

[B17] Fukada M. Hanai A. Nakayama A. Suzuki T. Miyata N. Rodriguiz R. M. (2012). Loss of Deacetylation activity of Hdac6 affects emotional behavior in mice. PLoS ONE 7:e30924 10.1371/journal.pone.0030924 22328923 PMC3273475

[B18] Gao Y. S. Hubbert C. Lu J. Lee Y. S. Yao T. P. (2007). Histone deacetylase 6 regulates growth factor-induced actin remodeling and endocytosis. Mol. Cell Biol. 27, 8637–8647 10.1128/MCB.00393-07 17938201 PMC2169396

[B19] Gauthier L. R. Charrin B. C. Borrell-Pages M. Dornpierre J. P. Rangone H. Cordelieres F. P. (2004). Huntingtin controls neurotrophic support and survival of neurons by enhancing BDNF vesicular transport along microtubules. Cell 118, 127–138 10.1016/j.cell.2004.06.018 15242649

[B20] Gines S. Paoletti P. Alberch J. (2010). Impaired, TrkB-mediated ERK1/2 activation in Huntington disease knock-in striatal cells involves reduced p52/p46 Shc expression. J. Biol. Chem. 285, 21537–21548 10.1074/jbc.M109.084202 20442398 PMC2898383

[B21] Hartmann D. Drummond J. Handberg E. Ewell S. Pozzo-Miller L. (2012). Multiple approaches to investigate the transport and activity-dependent release of BDNF and their application in neurogenetic disorders. Neural Plast. 2012:203734 10.1155/2012/203734 22720171 PMC3375105

[B22] Hartmann M. Heumann R. Lessmann V. (2001). Synaptic secretion of BDNF after high-frequency stimulation of glutamatergic synapses. EMBO J. 20, 5887–5897 10.1093/emboj/20.21.5887 11689429 PMC125691

[B23] Haubensak W. Narz F. Heumann R. Lessmann V. (1998). BDNF-GFP containing secretory granules are localized in synaptic junctions of cultured cortical neurons. J. Cell Sci. 111, 1483–1493 9580557 10.1242/jcs.111.11.1483

[B24] Hubbert C. Guardiola A. Shao R. Kawaguchi Y. Ito A. Nixon A. (2002). HDAC6 is a microtubule-associated deacetylase. Nature 417, 455–458 10.1038/417455a 12024216

[B25] Iwata A. Riley B. E. Johnston J. A. Kopito R. (2005). HDAC6 and microtubules are required for autophagic degradation of aggregated huntingtin. J. Biol. Chem. 280, 40282–40292 10.1074/jbc.M508786200 16192271

[B26] Kazantsev A. G. Thompson L. M. (2008). Therapeutic application of histone deacetylase inhibitors for central nervous system disorders. Nat. Rev. Drug Discov. 7, 854–868 10.1038/nrd2681 18827828

[B27] Kim C. Choi H. Jung E. S. Lee W. Oh S. Jeon N. L. (2012). HDAC6 inhibitor blocks amyloid beta-induced impairment of mitochondrial transport in hippocampal neurons. PLoS ONE 7:e42983 10.1371/journal.pone.0042983 22937007 PMC3425572

[B28] Kohara K. Kitamura A. Morishima M. Tsumoto T. (2001). Activity-dependent transfer of brain-derived neurotrophic factor to postsynaptic neurons. Science 291, 2419–2423 10.1126/science.1057415 11264540

[B29] Kolarow R. Brigadski T. Lessmann V. (2007). Postsynaptic secretion of BDNF and NT-3 from hippocampal neurons depends on calcium calmodulin kinase II signaling and proceeds via delayed fusion pore opening. J. Neurosci. 27, 10350–10364 10.1523/JNEUROSCI.0692-07.2007 17898207 PMC6673152

[B30] Lessmann V. Brigadski T. (2009). Mechanisms, locations, and kinetics of synaptic BDNF secretion: an update. Neurosci. Res. 65, 11–22 10.1016/j.neures.2009.06.004 19523993

[B31] Lessmann V. Gottmann K. Malcangio M. (2003). Neurotrophin secretion: current facts and future prospects. Prog. Neurobiol. 69, 341–374 10.1016/S0301-0082(03)00019-4 12787574

[B32] Li W. Calfa G. Larimore J. Pozzo-Miller L. (2012). Activity-dependent BDNF release and TRPC signaling is impaired in hippocampal neurons of Mecp2 mutant mice. Proc. Natl. Acad. Sci. U.S.A. 109, 17087–17092 10.1073/pnas.1205271109 23027959 PMC3479462

[B33] Martinowich K. Hattori D. Wu H. Fouse S. He F. Hu Y. (2003). DNA methylation-related chromatin remodeling in activity-dependent BDNF gene regulation. Science 302, 890–893 10.1126/science.1090842 14593184

[B34] Matsuda N. Lu H. Fukata Y. Noritake J. Gao H. Mukherjee S. (2009). Differential activity-dependent secretion of brain-derived neurotrophic factor from axon and dendrite. J. Neurosci. 29, 14185–14198 10.1523/JNEUROSCI.1863-09.2009 19906967 PMC3849773

[B35] Matsuyama A. Shimazu T. Sumida Y. Saito A. Yoshimatsu Y. Seigneurin-Berny D. (2002). *In vivo* destabilization of dynamic microtubules by HDAC6-mediated deacetylation. EMBO J. 21, 6820–6831 10.1093/emboj/cdf682 12486003 PMC139102

[B36] Nan X. CamPoy F. J. Bird A. (1997). MeCP2 is a transcriptional repressor with abundant binding sites in genomic chromatin. Cell 88, 471–481 10.1016/S0092-8674(00)81887-5 9038338

[B37] Ogier M. Wang H. Hong E. Wang Q. Greenberg M. E. Katz D. M. (2007). Brain-derived neurotrophic factor expression and respiratory function improve after ampakine treatment in a mouse model of Rett syndrome. J. Neurosci. 27, 10912–10917 10.1523/JNEUROSCI.1869-07.2007 17913925 PMC6672830

[B38] Park J. Cawley N. X. Loh Y. P. (2008). A bi-directional carboxypeptidase E-driven transport mechanism controls BDNF vesicle homeostasis in hippocampal neurons. Mol. Cell Neurosci. 39, 63–73 10.1016/j.mcn.2008.05.016 18573344 PMC2606928

[B39] Percy A. K. Lane J. B. (2005). Rett syndrome: model of neurodevelopmental disorders. J. Child Neurol. 20, 718–721 10.1177/08830738050200090301 16225824

[B40] Poo M. (2001). Neurotrophins as synaptic modulators. Nat. Rev. Neurosci. 2, 24–32 10.1038/35049004 11253356

[B41] Poon W. Blurton-Jones M. Tu C. H. Feinberg L. M. Chabrier M. A. Harris J. W. (2011). β -Amyloid impairs axonal BDNF retrograde trafficking. Neurobiol. Aging. 32, 821–833 10.1016/j.neurobiolaging.2009.05.012 19540623 PMC3038182

[B42] Porcher C. Hatchett C. Longbottom R. E. McAinch K. Sihra T. S. Moss S. J. (2011). Positive feedback regulation between gamma-aminobutyric acid type A (GABA(A)) receptor signaling and brain-derived neurotrophic factor (BDNF) release in developing neurons. J. Biol. Chem. 286, 21667–21677 10.1074/jbc.M110.201582 21474450 PMC3122223

[B43] Reed N. A. Cai D. Blasius T. L. Jih G. T. Meyhofer E. Gaertig J. (2006). Microtubule acetylation promotes kinesin-1 binding and transport. Curr. Biol. 16, 2166–2172 10.1016/j.cub.2006.09.014 17084703

[B44] Rivieccio M. A. Brochier C. Willis D. E. Walker B. A. D'Annibale M. A. McLaughlin K. (2009). HDAC6 is a target for protection and regeneration following injury in the nervous system. Proc. Natl. Acad. Sci. U.S.A. 106, 19599–19604 10.1073/pnas.0907935106 19884510 PMC2780768

[B45] Roux J. C. Zala D. Panayotis N. Borges-Correia A. Saudou F. Villard L. (2012). Modification of Mecp2 dosage alters axonal transport through the Huntingtin/Hap1 pathway. Neurobiol. Dis. 45, 786–795 10.1016/j.nbd.2011.11.002 22127389

[B46] Sadakata T. Shinoda Y. Oka M. Sekine Y. Sato Y. Saruta C. (2012). Reduced axonal localization of a Caps2 splice variant impairs axonal release of BDNF and causes autistic-like behavior in mice. Proc. Natl. Acad. Sci. U.S.A. 109, 21104–21109 10.1073/pnas.1210055109 23213205 PMC3529019

[B47] Tran A. D. A. Marmo T. P. Salam A. Che S. Finkelstein E. Kabarriti R. (2007). HDAC6 deacetylation of tubulin modulates dynamics of cellular adhesions. J. Cell Sci. 120, 1469–1479 10.1242/jcs.03431 17389687

[B48] Valenzuela-Fernandez A. Cabrero J. R. Serrador J. M. Sanchez-Madrid F. (2008). HDAC6: a key regulator of cytoskeleton, cell migration and cell-cell interactions. Trends Cell Biol. 18, 291–297 10.1016/j.tcb.2008.04.003 18472263

[B50] Wang X. Butowt R. Vasko M. R. von Bartheld C. S. (2002). Mechanisms of the release of anterogradely transported neurotrophin-3 from axon terminals. J. Neurosci. 22, 931–945 11826122 10.1523/JNEUROSCI.22-03-00931.2002 PMC6758481

[B51] Wang H. Chan S. A. Ogier M. Hellard D. Wang Q. Smith C. (2006). Dysregulation of brain-derived neurotrophic factor expression and neurosecretory function in Mecp2 null mice. J. Neurosci. 26, 10911–10915 10.1523/JNEUROSCI.1810-06.2006 17050729 PMC6674736

[B52] Zeev B. Bebbington A. Ho G. Leonard H. de Klerk N. Gak E. (2009). The common BDNF polymorphism may be a modifier of disease severity in Rett syndrome. Neurology 72, 1242–1247 10.1212/01.wnl.0000345664.72220.6a 19349604 PMC2677489

[B53] Zhang Y. Li N. Caron C. Matthias G. Hess D. Khochbin S. (2003). HDAC-6 interacts with and deacetylates tubulin and microtubules *in vivo*. EMBO J. 22, 1168–1179 10.1093/emboj/cdg115 12606581 PMC150348

[B54] Zhou Z. Hong E. J. Cohen S. Zhao W. N. Ho H. Y. Schmidt L. (2006). Brain-specific phosphorylation of MeCP2 regulates activity-dependent Bdnf transcription, dendritic growth, and spine maturation. Neuron 52, 255–269 10.1016/j.neuron.2006.09.037 17046689 PMC3962021

