# Supplementary figures and images for: A selective histone deacetylase-6 inhibitor improves BDNF trafficking in hippocampal neurons from Mecp2 knockout mice: implications for Rett syndrome
Source: Front Cell Neurosci. 2014 Mar 7;8:68. doi: 10.3389/fncel.2014.00068 (PMC3945638; doi:10.3389/fncel.2014.00068)

**A**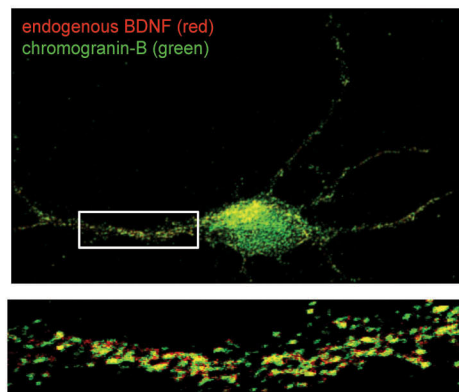**B**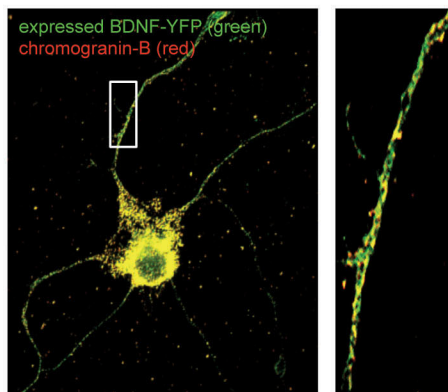**C****WT**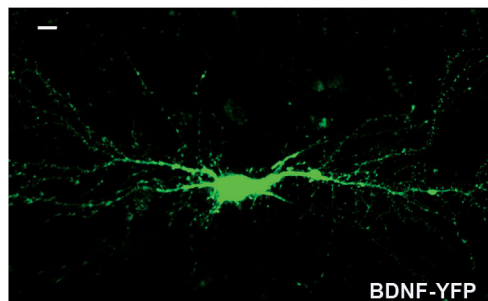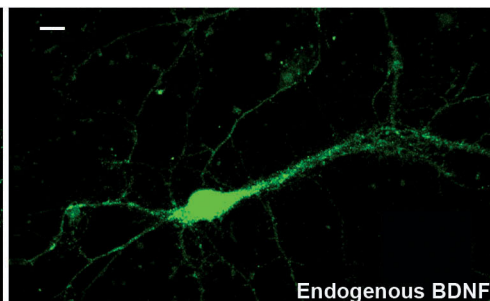**D*****Mecp2*<sup>-/-</sup>**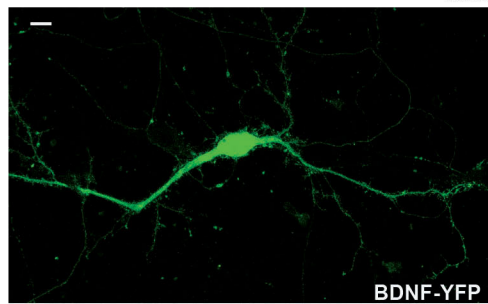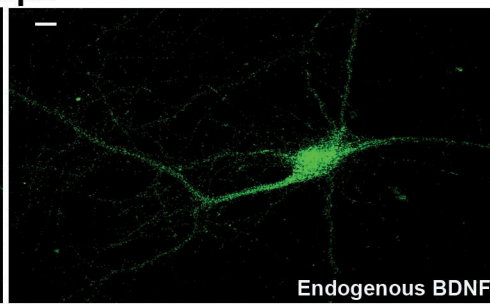

Supplement: Supplemental Figure 1 — Distribution of exogenously expressed BDNF-YFP is comparable to that of endogenous native BDNF. (A) Representative example of a cultured hippocampal pyramidal neuron after dual immunostaining for endogenously expressed native BDNF (red) with chromogranin-B (green), a marker of secretory granules. The inset shows colocalization in a dendritic segment. (B) Immunostaining for chromogranin-B (red) on a BDNF-YFP-expressing neuron (green) reveals essentially the same patter of colocalization. (C,D) The same pattern of co-localization between exogenous BDNF-YFP and endogenous BDNF is observed in cultured hippocampal pyramidal neurons from either WT (C) mice or Mecp2 knockout mice (D). Scale bar = 10 μm. [file Presentation1.PDF]

**A**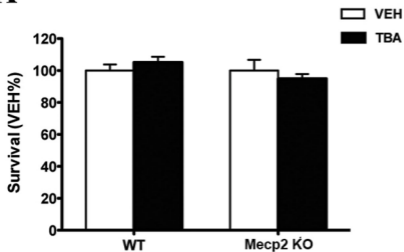**B**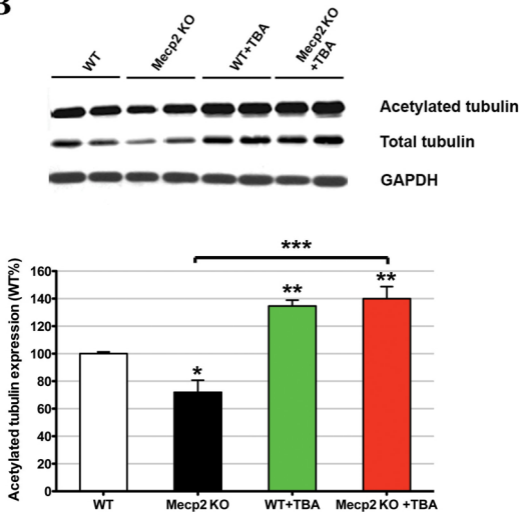

Supplement: Supplemental Figure 2 — TBA increases tubulin acetylation. (A) Cell viability assessed by trypan blue exclusion. (B) Extracts from DIV12 wildtype cells and Mecp2 knockout neurons treated with or without TBA (1 μ M) for 48 h were processed by Western blot and analyzed for acetylated tubulin and total tubulin. GAPDH was performed as loading control (top). Quantification of protein levels for acetylated tubulin in each group (n = 4) was normalized to that in wildtype neurons (WT) and expressed as % of WT. *p < 0.05; **p < 0.01 compared to WT group; ***p < 0.001 compared to Mecp2 knockout group (bottom). [file Presentation2.PDF]
